# Structural Basis of Differential Neutralization of DENV-1 Genotypes by an Antibody that Recognizes a Cryptic Epitope

**DOI:** 10.1371/journal.ppat.1002930

**Published:** 2012-10-04

**Authors:** S. Kyle Austin, Kimberly A. Dowd, Bimmi Shrestha, Christopher A. Nelson, Melissa A. Edeling, Syd Johnson, Theodore C. Pierson, Michael S. Diamond, Daved H. Fremont

**Affiliations:** 1 Department of Pathology & Immunology, Washington University School of Medicine, Saint Louis, Missouri, United States of America; 2 Viral Pathogenesis Section, Laboratory of Viral Diseases, National Institute of Allergy and Infectious Diseases, National Institutes of Health, Bethesda, Maryland, United States of America; 3 Department of Medicine (Infectious Diseases), Washington University School of Medicine, Saint Louis, Missouri, United States of America; 4 MacroGenics, Rockville, Maryland, United States of America; 5 Department of Molecular Microbiology, Washington University School of Medicine, Saint Louis, Missouri, United States of America; 6 Department of Biochemistry and Molecular Biophysics, Washington University School of Medicine, Saint Louis, Missouri, United States of America; Institut Pasteur, France

## Abstract

We previously developed a panel of neutralizing monoclonal antibodies against Dengue virus (DENV)-1, of which few exhibited inhibitory activity against all DENV-1 genotypes. This finding is consistent with reports observing variable neutralization of different DENV strains and genotypes using serum from individuals that experienced natural infection or immunization. Herein, we describe the crystal structures of DENV1-E111 bound to a novel CC′ loop epitope on domain III (DIII) of the E protein from two different DENV-1 genotypes. Docking of our structure onto the available cryo-electron microscopy models of DENV virions revealed that the DENV1-E111 epitope was inaccessible, suggesting that this antibody recognizes an uncharacterized virus conformation. While the affinity of binding between DENV1-E111 and DIII varied by genotype, we observed limited correlation with inhibitory activity. Instead, our results support the conclusion that potent neutralization depends on genotype-dependent exposure of the CC′ loop epitope. These findings establish new structural complexity of the DENV virion, which may be relevant for the choice of DENV strain for induction or analysis of neutralizing antibodies in the context of vaccine development.

## Author Summary

Within each Dengue virus (DENV) serotype, viruses are subdivided into genotypes based upon the protein sequence variation. Infection with a given serotype is believed to induce neutralizing antibodies that provide long-term immunity against secondary infection by a strain of the same serotype. However, recent studies suggest that some classes of neutralizing antibodies fail to inhibit infection equivalently for all genotypes within a DENV serotype. DENV1-E111 is an example of an antibody that differentially neutralizes infection of DENV-1 strains. We used structural and molecular approaches to determine that DENV1-E111 binds to an epitope in domain III of the envelope protein. Although the epitope sequence varied between DENV-1 genotypes, inhibitory activity of the antibody remained unequal when we exchanged the amino acids within the epitope among genotypes. Docking of our structures onto DENV virion models revealed that the DENV1-E111 epitope was inaccessible, suggesting that the antibody recognizes an uncharacterized virus conformation. Our studies suggest that DENV virion structures differ in a genotype-dependent manner, which can impact the inhibitory activity of antibodies that recognize cryptic epitopes.

## Introduction

Dengue viruses (DENV) are mosquito-borne viruses of the Flavivirus genus, which include other significant human pathogens such as West Nile (WNV), Japanese encephalitis, and yellow fever viruses. Infection with DENV can cause symptoms in humans ranging from a mild febrile illness (Dengue Fever, DF) to a more severe hemorrhagic fever (DHF) and life-threatening dengue shock syndrome (DSS). Currently, it is estimated that DENV infects ∼50 to 100 million people per year resulting in ∼250,000 to 500,000 cases of DHF/DSS [1]. The four serotypes of DENV (DENV-1, DENV-2, DENV-3, and DENV-4) comprise a genetically related yet antigenically distinct serocomplex, varying from one another by 25 to 40% at the amino acid level. Each DENV serotype is further divided into genotypes, which can vary up to 3% [2], [3]. Currently, there are no specific antiviral therapies or vaccines approved for use in humans, and treatment of severe disease remains supportive in a tertiary care setting.

The humoral response contributes to protection and also, paradoxically to the pathogenesis of severe DENV disease. Infection with a given serotype is believed to induce durable levels of neutralizing antibodies that provide life-long immunity against subsequent challenge by a strain of the same serotype [4]. However, secondary infection with a heterologous serotype increases the relative risk of developing DHF and DSS [5]. A favored hypothesis is that during secondary infection poorly neutralizing cross-reactive antibodies from the primary infection enhance infection of the heterologous virus in cells bearing Fc-γ receptors [6]. Recent studies in non-human primates and mice have confirmed that passive transfer of anti-DENV monoclonal or polyclonal antibodies can augment replication of a heterologous DENV in challenge models, and in some cases cause a lethal vascular leakage syndrome that resembles DSS [7]–[9].

Humoral protection against DENV correlates with the induction of a neutralizing antibody response against the envelope (E) protein on the surface of the virion ([10] and reviewed in [11]). The ectodomain of the DENV E protein is composed of three domains [12]: DI is a central nine-stranded β-barrel domain, DII consists of two finger-like protrusions from DI and contains the hydrophobic peptide required for virus fusion, and DIII is an immunoglobulin-like domain on the other side of DI that has been proposed to interact with as yet uncharacterized host receptor(s). Neutralizing monoclonal antibodies (MAbs) against the different DENV serotypes map to all three domains [13]–[19], although many potently inhibitory mouse MAbs localize to DIII [13]. To date, three epitopes have been established on DIII [7], [20], [21]: MAbs binding the lateral ridge or A-strand epitope are relatively inhibitory, whereas MAbs recognizing the AB loop neutralize infection less efficiently or not at all [22], presumably due to poor epitope accessibility on the virion.

Cryo-electron microscopy (cryo-EM) studies have revealed that the E proteins of mature flavivirus virions form anti-parallel dimers that lie flat against the surface of the virion and are arranged with T = 3 quasi-icosahedral symmetry [23], [24]. In this configuration, E proteins exist in three distinct chemical environments defined by their proximity to the 2-, 3-, or 5-fold axis of symmetry [23], [24]. While 180 copies of the E protein are present on all flavivirus virions, the different environments imposed by the quasi-icosahedral symmetry make some epitopes unequally accessible. Epitope exposure also may be affected by neighboring E proteins in adjacent symmetry units, or by the presence or absence of prM in the case of immature, partially mature, and mature virions [25]–[30]. The arrangement of the E proteins on the surface of the virion can be modulated over time and across a range of temperatures due to the intrinsic conformational heterogeneity of virions [31], [32] Consequently, the accessibility of epitopes can vary across structurally distinct epitopes, ultimately affecting the number of sites available for antibody binding and neutralization.

Recently, we generated a panel of 79 MAbs against DENV-1 to define how antibodies neutralized different DENV-1 genotypes [17]. Within this panel, 15 MAbs were potently neutralizing, and most mapped to previously identified epitopes in DIII, although few retained strong inhibitory activity against heterologous DENV-1 genotypes. Prior studies have described disparate neutralization of DENV strains corresponding to different genotypes within a serotype with serum from natural infection [33]–[35] or after immunization with live-attenuated tetravalent vaccine candidates [34], [36]–[38]. One such DIII-specific neutralizing MAb from our panel, DENV1-E111 (henceforth termed E111) potently neutralized infection of a genotype 2 DENV-1 (strain 16007, EC50 of ∼4 ng/ml), but inhibited infection of a genotype 4 virus poorly (strain Western Pacific-74 (West Pac-74), EC50 of ∼15,200 ng/ml). Sequence analysis of the variation between residues 296–400 of DIII for 16007 or West Pac-74 revealed only two differences (amino acids 339 and 345), with amino acid 345 as the only residue that varied in all five DENV-1 genotypes.

Here, we determined the crystal structures of an E111 single-chain variable fragment (scFv) in complex with DIII of 16007 and the E111 Fab in complex with DIII from West Pac-74. E111 bound to a previously uncharacterized epitope centered on the CC′ loop of DIII, which should not be exposed on the virion according to existing flavivirus atomic models; our structural data defining the CC′ loop epitope of E111 was supported by extensive mutagenesis and binding analyses. While E111 showed a higher affinity and longer half-life of binding to DIII of 16007 (genotype 2) compared to DIII from several other DENV-1 genotypes, this did not explain the disparity in neutralization potency for viruses from all five genotypes. Mutation at position 345 of West Pac-74 DIII to the corresponding residue in 16007 resulted in increased E111 binding, but only a small improvement in neutralization potency, suggesting that differences in amino acids within the epitope among genotypes could not account for the phenotype. However, neutralization of DENV-1 West Pac-74 with E111 was enhanced by incubating virus-antibody complexes at higher temperature or for longer times, whereas this treatment failed to equivalently impact inhibition of strain 16007 by E111. Our experiments suggest that the conformational ensemble of DENV virion structures differs in a genotype-dependent manner, which impacts the neutralizing activity of antibodies that recognize nominally cryptic epitopes.

## Results

### Structure of E111 in complex with DIII

We initially formed complexes of E111 Fab with soluble, bacterially expressed DIII (residues 293–399) cloned from 16007 and West Pac-74 DENV-1 strains (**Figure S1**, and data not shown). Several conditions yielded diffracting crystals of the 16007 DIII-E111 Fab complex but failed to diffract better than ∼6.0 Å resolution. As an alternative strategy, we cloned the heavy (V_H_) and light chain (V_L_) variable domain sequences from the E111 hybridoma to create an scFv. Two constructs of the E111 scFv were generated, with either the V_L_ or V_H_ sequence at the N-terminus, separated with a (GGGGS)_3_ linker, and a C-terminal hexahistadine tag for affinity purification. These inserts were cloned into the pAK400 vector that contains a pelB leader sequence for targeting the polyprotein transcript to the oxidative environment of the bacterial periplasm [39]. Sequential purification by nickel affinity and size exclusion chromatography revealed two species of scFv: a non-disulfide domain-swapped dimer and a monomer. For our structural analysis, we used the monomeric species with V_L_ at the N-terminus (**Figure S1A**).

**Figure 1:**
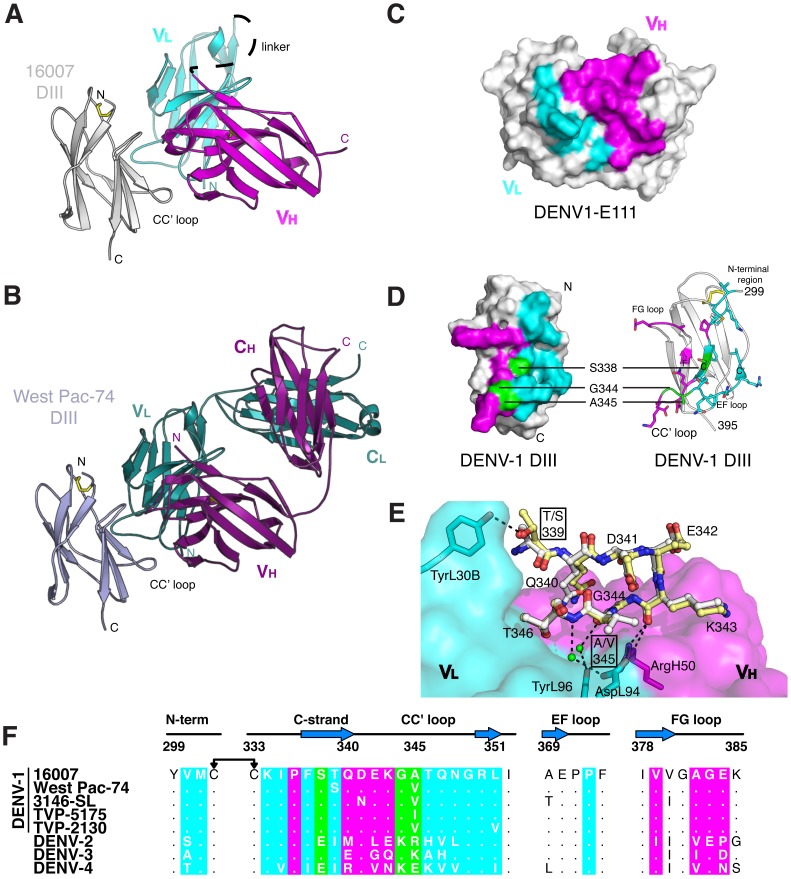
E111 in complex with DENV-1 DIII. A ribbon diagram of the crystal structure of (**A**) E111 scFv in complex with DENV-1 strain 16007 DIII and (**B**) E111 Fab in complex with DENV-1 strain West Pac-74 DIII. The light chain is colored in cyan and the heavy chain in magenta. 16007 DIII is colored in light gray and West Pac-74 DIII is in gray. (**C**) A surface model showing the contact residues in the scFv complex. Contacts in the scFv are highlighted by light chain (cyan) or heavy chain (magenta) contacts. DIII contacts are highlighted by heavy chain (magenta), light chain (cyan), or both chains (green). (**D**) A ribbon diagram showing the residues of DIII contacted by the E111 scFv in the crystal structure. DIII residues that are contacted by the heavy and light chains of E111 (338, 344, and 345) are labeled. (**E**) A close-up of the contacts made by E111 in the CC′ loop of 16007 DIII (yellow) or West Pac-74 DIII (white). (**F**) Sequence of the four segments of 16007 DIII contacted by E111 aligned with the analogous residues of the other four DENV-1 genotypes and three serotypes. Residues are colored as in **Figure 1D**.

We determined the structure of E111 scFv in complex with DIII of DENV-1 16007 to 2.5 Å resolution (model statistics are in **Table 1**). There were no major structural perturbations to the immunoglobulin-like β-sandwich topology of DIII found in other flavivirus E proteins, with a root mean squared displacement of 0.7 Å compared to unbound DIII. The E111 scFv adopted the predicted variable domain assembly (**Figure 1A**). The binding interface had an average degree of shape complementarity (*S*_c_ = 0.68, with *S*_c_ = 1.0 a perfect score) for antibody-antigen interactions and 2,095 Å^2^ of combined surface area. The light and heavy chains engaged DIII equivalently, with a combined buried surface area of 1,017 Å^2^ (460 Å^2^ of DIII and 557 Å^2^ of the light chain) versus 1,078 Å^2^ (550 Å^2^ of DIII and 528 Å^2^ of the heavy chain) (**Figure 1C**). The interaction between E111 and DIII of strain 16007 was dominated by hydrophobic interactions, in addition to six direct hydrogen bonds and fourteen water-mediated networks at the interface of the complex (**Table S1**).

**Table 1:** Data collection and refinement statistics.

Data collection		
	16007 DIII	Western Pacific-74 DIII
	E111 scFv	E111 Fab
Space group	P4_3_2_1_2	P2_1_
Cell dimensions (Å)	a = b = 135.2, c = 52.2	a = 82.9, b = 52.0, c = 136.4
Total reflections	105832	44787
Unique reflections	17132	11029
Resolution	50.0-2.5 Å (2.59-2.50)	50.0-3.8 Å (3.97-3.8)
Completeness	98.1% (94.3%)	97.4% (98.6%)
*R*_sym_	9.2% (49.4%)	12.0% (51.4%)
I/σ (I)	18.78 (2.35)	9.74 (1.91)

Refinement		
Resolution	41.36-2.50 Å (2.65-2.50)	43.45-3.79 Å (4.18-3.80)
R-work	17.2%	23. 7%
R-free	22.9%	27.8%
Ramachandran favored	97.4%	97.0%
Ramachandran outliers	0.3%	0.4%
Rotamer outliers	0%	0.0%
Protein residues	328	1060
Water molecules	139	Not Placed
R.m.s.d. bond angles	0.004°	0.003°
R.m.s.d. bond lengths	0.883 Å	0.860 Å
MolProbity score	100^th^ percentile	100^th^ percentile
MolProbity clash score	99^th^ percentile	97^th^ percentile

Values in parentheses are for the highest resolution shell.

We obtained crystals of DIII of West Pac-74 in complex with E111 Fab that diffracted to 3.8 Å resolution (**Figure S1B**). There were two complexes in the asymmetric unit, and non-crystallographic symmetry restraints were applied in the refinement (model statistics are in **Table 1**). The two Fabs have essentially identical structures with the notable exception of the elbow angles between the variable and constant domains ((149.0° versus 133.5° as calculated by the RBOW server [40]). As with the co-crystal structure with the E111 scFv and DIII of 16007, there were limited conformational changes in the West Pac-74 DIII upon E111 Fab ligation (R.m.s.d = 0.8 Å). The E111 Fab-DIII interface also had a similar average degree of shape complementarity (*S*_c_ = 0.65) and total buried surface area (2,076.5 Å^2^). Overall, the E111 scFv and Fab structures (DIII and Fv domains) varied little from one another in terms of structure (R.m.s.d = 0.3 Å) or orientation of engagement of DIII (**Figure S1C**).

E111 engaged discontinuous segments of DIII of 16007 and West Pac-74 including the N-terminal linker (residues 300–301), C-strand, CC′ loop, C′-strand (residues 334–351), EF loop (372), and FG loop (residues 382–384) (**Figure 1D**); together, these form a single convex surface patch of 25 residues. A total of 20 residues of E111 contacted DIII: 8 from the light chain and 12 from the heavy chain. The heavy and light chains both formed contacts with three of the same amino acid residues (S338, G344, and A345). The E111 binding site was centered on the CC′ loop (7 of 25 residues), a previously uncharacterized epitope for flavivirus neutralizing MAbs (**Figure 1E**). Analysis of the CC′ loop sequences from other DENV serotypes revealed significant variation (**Figure 1F**), which likely explains the type-specificity (i.e., does not bind or neutralize other DENV serotypes) of E111 [17].

We previously observed reduced binding of E111 to DENV-1 West Pac-74 strain (genotype 4) in a virus capture ELISA, which correlated with a ∼4,100-fold decrease in neutralization efficiency [17]. E111 contacted every residue in the CC′ loop of 16007 DIII, as well as residues on the adjacent C- and C′-strands. Sequence variation between 16007 (genotype 2) and West Pac-74 (genotype 4) occurs at two DIII positions, 339 and 345, both of which are directly contacted by E111.

### Binding of mutant DIII to E111

**Figure 2:**
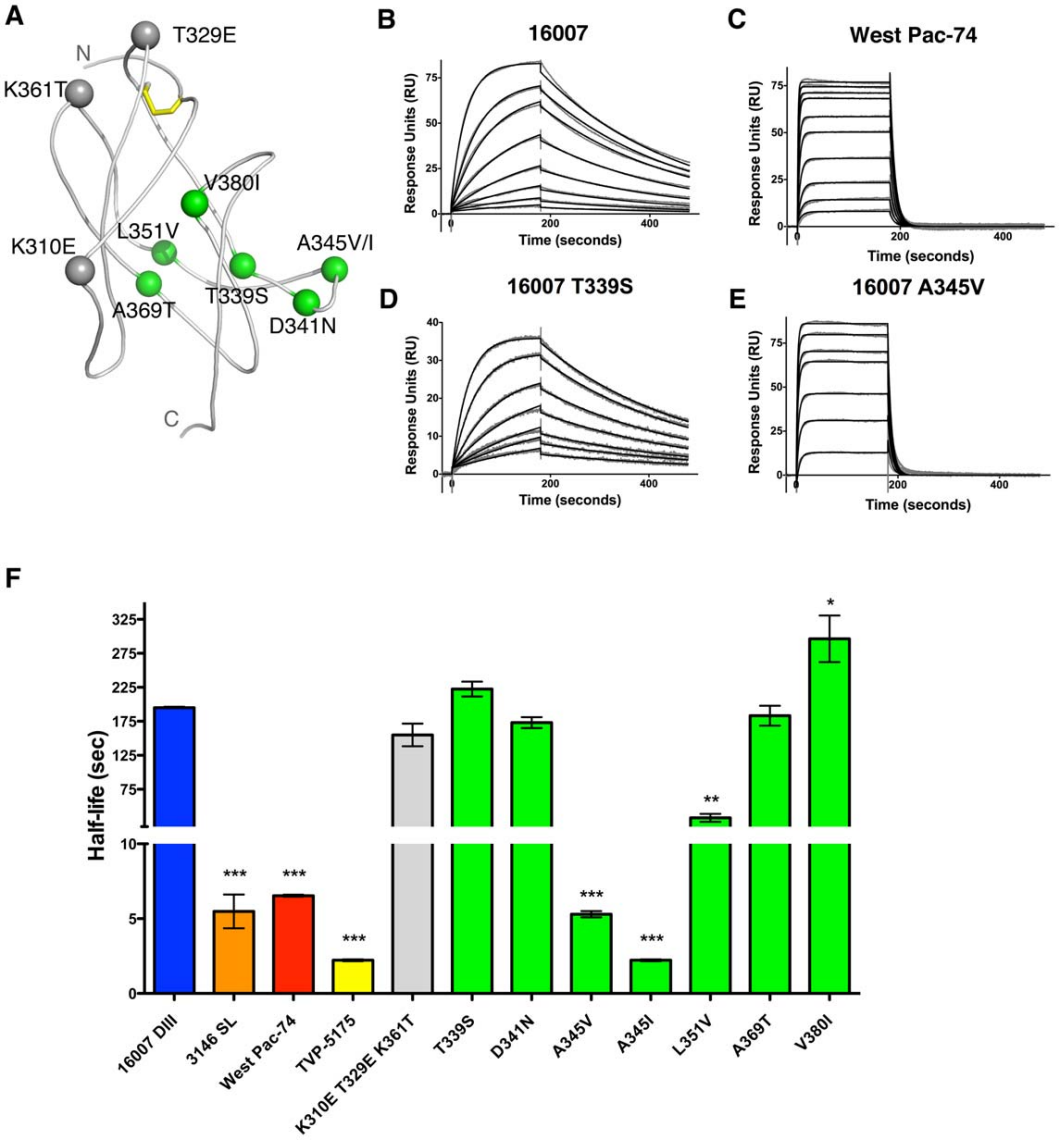
Kinetic analysis of E111 interaction with DENV genotypes and single variants. (**A**) Ribbon diagram of DENV-1 16007 DIII with amino acids highlighted corresponding to tested mutants. Lateral ridge mutants and A-strand mutants are shown in gray. Amino acids representing genotypic variation of DENV-1 are shown in green. **B–E**. SPR traces are presented of E111 MAb interacting with (**B**) 16007 DIII, (**C**) West Pac-74 DIII, (**D**) 16007 DIII containing a T339S substitution or (**E**) 16007 DIII containing an A345V substitution. A single representative sensogram is shown for each DIII variant. The experimental curves (*gray lines*) were fit using a 1∶1 Langmuir analysis (*black lines*), after double referencing, to determine the kinetic parameters presented in **panel F and** **Table 2**. Graphical representation of E111 binding to 16007 DIII mutants is presented according to the half-life (in seconds) of the interaction. The results are representative of a minimum of three independent experiments, with error bars showing standard deviation. Statistical significance was determined using a paired student t-test comparing the half-life of the E111 binding to 16007 DIII to that of another DIII variant.

As variation within the CC′ loop and surrounding regions might affect the differential binding and neutralization of E111 for different DENV-1 genotypes, we generated a library of soluble DENV-1 DIII proteins based upon natural sequence variation of all five DENV-1 genotypes and tested their binding kinetics at 25°C to E111 by surface plasmon resonance (SPR) (**Figure 2A, 2F** **, and** **Table 2**). Whereas E111 had a *K*_D_ of 18.0±0.08 nM and a half-life of 194 seconds for DIII from 16007, its interaction with West Pac-74 DIII was weaker with a *K*_D_ of 415±24 nM and half-life of 6.5 seconds (**Figure 2B and 2C**). Mutagenesis of a T→S at position 339 had a similar affinity as wild type 16007 DIII (*K*_D_ of 16.6±0.17 nM; t_1/2_ = 222.4 seconds (**Figure 2D**)). The crystal structures show that the additional methyl group present in the 16007 Thr residue does not contact E111, whereas the Ser/Thr hydroxyl groups both make equivalent hydrogen bonds with TyrL30B in CDR1 of the E111 light chain (see **Figure 1E**). In contrast, an A→V change at position 345 of 16007 DIII (to the residue in West Pac-74) decreased the affinity of binding such that kinetics became comparable to West Pac-74 DIII with a *K*_D_ of 1143±60 nM and half-life of 5.3 seconds (**Figure 2E**). The side chain of Ala 345 of 16007 makes limited contact with E111, and the additional two methyl groups in Val 345 of West Pac-74 appear to be tolerated sterically at the E111 interface with only minor structural perturbations. However, position 345 and the adjacent CC′ loop residues participate in an extensive network of hydrogen bonds with E111 (**Figure S1D**), and we speculate that Val 345 leads to a dramatically faster off-rate by subtle destabilization of this interface. Due to the low resolution of the E111 Fab-West Pac-74 DIII structure, ordered water molecules could not be modeled, making precise comparison of the interfaces difficult.

**Table 2:** SPR results for binding of DENV-1 E111 to DIII variants.

DIII variant	Binding parameters for DENV1-E111^a^					
	ka (10^5^M-1s-1)	kd (10^−3^s-1)	*K*_D_ (nM)	?2	*t*1/2 (sec)	*t* ½ significance^b^
16007	1.98±0.09	3.56±0.03	18±0.08	1.4±0.6	194.1±1.5	-
Western Pacific-74	2.56±0.18	106.4±2	416±24	2.4±1.1	6.5±0.1	***
3146 SL	2.67±1.02	141.6±64	523±32	3.6±0.8	5.5±2.0	***
TVP-5175	1.62±0.28	312.3±13.6	1981±441	0.2±0.08	2.2±0.1	***
16007 K310E/T329E/K361T	1.21±0.4	4.57±0.77	39.8±0.9	0.29±0.2	154.9±29	N.S.
16007 T339S	1.91±0.4	3.13±0.27	16.6±0.17	0.75±0.6	222.4±19	N.S.
16007 D341N	2.13±0.2	4.02±0.3	18.9±0.06	0.63±0.08	173.1±14	N.S.
16007 A345V	1.14±0.003	131.0±8.8	1143±60	3.3±1.8	5.3±0.4	***
16007 L351V	2.11±0.7	23.7±0.8	107.2±0.01	0.23±0.1	32.9±11	**
16007 A369T	1.51±0.12	3.83±0.5	25.3±0.1	0.53±0.3	182.3±25	N.S.
16007 V380I	1.24±0.05	2.36±0.3	19.0±0.2	0.45±0.5	296.4±34	*
E111 scFv-16007 DIII	2.88±0.7	6.95±0.7	24.6±3.5	0.217±0.09	100.5±9.7	**
E111 scFv- West Pac-74 DIII	1.51±0.08	275±21.9	1830±169	0.31±0.08	2.53±0.2	***

a Values for ka, kd, *K*_D_ are means ± standard deviations. *K*_D_ = kd/ka; t1/2 = ln(2)/kd.

b *P*-values were determined in comparison to the 16007 half-life value by t-test:

* *P*<0.05,

** *P*<0.01,

*** *P*<0.001.

While the on-rates for E111-DIII interactions were relatively constant, the off-rate governed the differences in affinity for the DIII variants (**Figure 2F**). DIII from strain 3146 SL varies in six positions from 16007, including a valine at position 345. Kinetic analysis with 3146 SL DIII revealed a decreased half-life (5.5 seconds) as compared to 16007. Substitution of individual amino acids corresponding to variation in strain 3146L (D341N (CC′ loop) or A369T (E-strand)) had little negative effect on the half-life of strain 16007 (t_1/2_ of 173 seconds and 182 seconds, respectively). One amino acid difference (V380I (F-strand)) in 3146 SL caused a small increase in the half-life (t_1/2_ of 296 seconds) when inserted into DIII of 16007, despite its side chain location ∼10 Å from DIII. An A→I change at position 345 was the only difference in DIII sequence between strains 16007 (genotype 2) and TVP-5175 (genotype 3); this single amino acid substitution reduced the half-life (t_1/2_ of 2.2 seconds) and affinity of binding (*K*_D_ of 1981±441 nM). Precise kinetic measurements with DIII from TVP-2130 (genotype 1) were limited by non-specific interactions, and thus not analyzed (data not shown). However, a DIII variant of 16007 that included the unique variation of strain TVP-2130 in the CC′ loop (L351V) showed a decreased half-life with E111 (t_1/2_ of 32.9 seconds) likely due to the disruption of optimal hydrophobic contact with Tyr30B of the variable light chain CDR1 loop. As a control, insertion of a triple mutation of K310E/T329E/K361T into the 16007 DIII lateral ridge and A-strand epitopes did not alter significantly E111 binding affinity or half-life (t_1/2_ of 155 seconds). SPR binding studies with the E111 scFv showed a similarly reduced half-life for West Pac-74 DIII compared to 16007 DIII (**Table 2**). A similar kinetic pattern of E111-DIII interactions also was observed at 37°C (data not shown). Overall, our genetic and biophysical studies support the crystallographic analysis and establish the CC′ loop as important for recognition of DIII by E111.

### Differential neutralization of DENV-1 genotypes by E111

**Figure 3:**
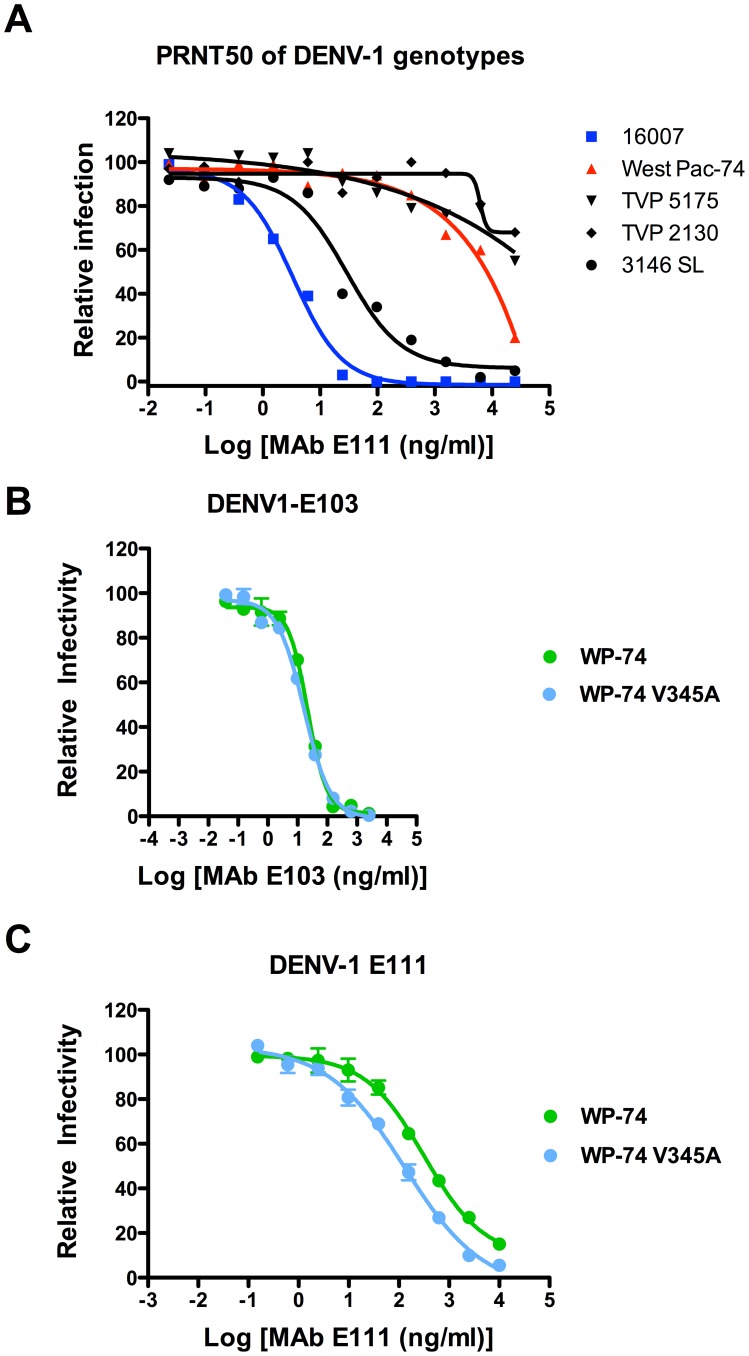
E111 neutralization of different DENV-1 genotypes is only partially dependent on differences in epitope sequence. (**A**) Plaque reduction and neutralization curves for five DENV-1 strains representing the five genotypes. The data is representative of three independent experiments performed in duplicate. PRNT50 values are shown in **Table 3**. **B–C**. Serial six-fold dilutions of (**B**) DENV1-E103 or (**C**) E111 were incubated with wild type or V345A West Pac-74 RVPs for one hour at 37°C, and then added to Raji-DC-SIGNR cells. Infection was assessed by flow cytometry 48 hours later. One representative experiment of four is shown. The data is normalized relative to the infectivity of the RVPs in the absence of antibody. Error bars indicate standard error of the mean of replicate infections.

We determined the inhibitory activity of E111 against strains representing the three other genotypes of DENV-1: TVP-2130 (genotype 1), TVP-5175 (genotype 3), and 3146 SL (genotype 5). Only strains corresponding to genotypes 2 and 5 (16007 and 3146 SL) were neutralized potently by E111 with EC50 values of 3.8±2.0 ng/ml and 22±10 ng/ml, respectively (**Figure 3A** **and** **Table 3**). In comparison, E111 neutralized strains of genotype 1, 3, and 4 poorly with EC50 values ranging from 9,700±3,200 ng/ml to greater than 25,000 ng/ml. Although rather extreme differences in E111-mediated neutralization were observed with different genotypes, this pattern failed to correlate with the *K*_D_ or half-life of binding with recombinant DIII by SPR (see **Table 2**). These results suggest that DIII epitope sequence-independent factors (e.g., CC′ epitope accessibility on the virion or secondary binding sites in other domains) likely contribute to the differential genotype neutralization by E111.

**Table 3:** PRNT50 values of E111 against strains representing five DENV-1 genotypes.

DENV-1 strain	Genotype	PRNT50 (ng/ml) ± SD
16007	2	3.8 (±2.0)
3146 SL	5	21 (±10)
Western Pacific-74	4	9,720 (±3,180)
TVP-5175	3	>25,000 ng/ml
TVP-2130	1	>25,000 ng/ml

Neutralizing activity was determined by PRNT assay on Vero cells with increasing concentrations of purified DENV1-E111 and 10^2^ PFU of the indicated DENV-1 genotypes. The data was derived from three independent experiments performed in duplicate. PRNT50 values were calculated by non-linear regression analysis and SD indicates the standard deviations.

### Substitution of V345A into the West Pac-74 strain only modestly enhances neutralizing activity of E111

Our SPR data demonstrated that substitution of a single residue (A→V) at position 345 of soluble 16007 DIII reduced the E111 binding half-life to that observed with DIII of West Pac-74. To test the effect of a reciprocal V→A change at position 345 in the West Pac-74 strain, we used a reverse genetic system: DENV-1 West Pac-74 reporter virus particles (RVP) [32], [41] incorporating a single V345A mutation were analyzed for sensitivity to MAb neutralization. Whereas DENV1-E103 MAb (which maps to residues T303, G328, T329, D330, and P332 on the lateral ridge of DIII [17]) neutralized both wild-type and V345A DENV-1 West Pac-74 RVP equivalently (**Figure 3B**), E111 showed only moderately enhanced neutralization of the V345A-containing RVP (3.6-fold, *P*<0.05, **Figure 3C**). Although the V345A change improved neutralization of West Pac-74 by E111, it failed to restore the sensitivity seen with DENV-16007 RVP or the fully infectious virus. Thus, either additional amino acid residues accounted for the genotypic difference in neutralization or the epitope was not displayed equivalently on the two viruses.

### The structural basis of E111 neutralization

**Figure 4:**
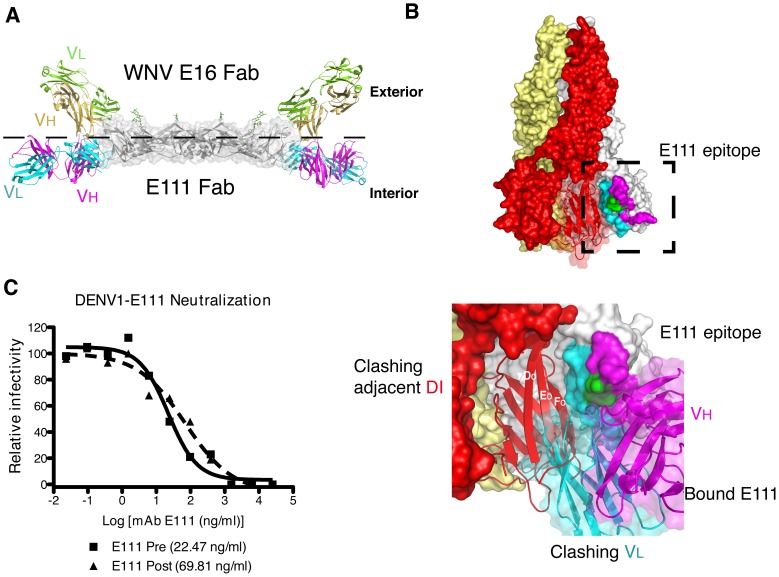
The structural basis of E111 neutralization. (**A**) E111 Fab docked onto the DENV-2 dimer (PDB 1OAN). Transparent gray space filled-ribbon model of DENV-2 dimer with N-linked glycans colored green. The E111 Fab light (*cyan*) and heavy (*magenta*) chains are shown bound to the equivalent DIII of the three dimensional structure within the DENV-2 dimer. The WNV E16 Fab (DIII lateral ridge antibody), in green and gold, (PDB 1ZTX) is docked onto the analogous DENV-2 residues for comparison. (**B**) Surface representation of the DENV-1 post-fusion trimer structure. *Upper panel*: Each E protein in the trimer is colored independently (white, red and yellow), and the E111 epitope (colored as in **Figure 1D**) is mapped onto DIII. The red surface of DI was made transparent to show the ribbon structure. *Lower panel*: The E111 scFv complex was superimposed onto a monomer of the post-fusion trimer (white); The E111 scFv light chain (cyan) bound to DIII clashes with DI of the neighboring E monomer (red) suggesting that E111 likely would inhibit formation of the E homotrimer required for virus fusion. Clashing beta strands from DI are labeled. (**C**) To determine whether E111 neutralizes infection before or after cellular attachment, BHK21-15 cells were pre-chilled to 4°C, and 10^2^ PFU of DENV-1 (16007) was added to each well for 1 h at 4°C. After extensive washing at 4°C, increasing concentrations of E111 were added for 1 h at 4°C, and the PRNT protocol was then completed (dashed lines, *Post*). In comparison, a standard pre-incubation PRNT with all steps performed at 4°C is shown for reference. In this case, virus and MAb were incubated together for 1 h at 4°C, prior to addition to cells (solid lines, *Pre*). Data shown are representative from three experiments performed in duplicate.

Because of the differential neutralization of West Pac-74 and 16007 by E111, we evaluated its epitope in the context of full-length E protein structures. We docked our scFv–DIII complex onto the available structure of the pre-fusion DENV E protein dimer (PDB ID 1OAN [12]), and compared this to other characterized DIII-specific anti-flavivirus neutralizing MAbs (**Figure 4A**). E111 engaged the face of DIII opposite to the one seen previously with 1A1D-2 and 4E11 (A-strand) [31], [42] or WNV-E16 (lateral ridge) [25]. The E111 Fab was rotated in a downward orientation compared to the WNV E16 Fab (**Figure 4A**) or the A-strand DENV Fabs docked onto the same structure (data not shown). Based on this docking it appears that E111 does not bind the outer exposed surface of the DENV-1 E protein but rather a determinant that is localized to the interior of the virus.

Antibody neutralization of flaviviruses can occur by blocking attachment, internalization, and/or endosomal fusion. Prior to viral fusion, the E proteins on the surface of the virus dissociate from their dimeric hairpin arrangement to form trimeric spikes upon acidification in the late endosome. This rearrangement is essential to allow the newly exposed fusion loop to insert into the endosomal membrane. While the exact structural transitions of the E proteins from dimer to trimer remain unknown, DIII is displaced ∼70° from the pre-fusion structure and settles adjacent to DI in the post-fusion state [43]–[45]. We examined structurally how E111 could disrupt a post-attachment step by docking our Fab-DIII complex onto the structure of the DENV-1 post-fusion trimer [45]. While the Fab does not clash with the adjacent E protein in the DENV-2 pre-fusion dimer structure (**Figure 4A**), the light chain of E111 sterically would inhibit formation of the post-fusion trimer (**Figure 4B**) by clashing with the neighboring DI of an adjacent E protein. Thus, from a structural perspective, E111 likely hinders the necessary conformational change from E protein homodimer to homotrimer, and limits viral fusion and infection.

To begin to understand the mechanism of E111-mediated neutralization, we performed pre- and post-attachment neutralization assays [10], [18], [25], [46]. E111 MAb was incubated with DENV-1 16007 before or after virus binding to BHK21-15 cells, and infection was measured by the plaque reduction assay. E111 efficiently neutralized DENV-1 when premixed with the virus before cell attachment or when added after the virus had attached to the cell surface (**Figure 4C**). This result suggests that E111 has the capacity to neutralize infection after virus attachment has occurred, and is consistent with previously observed patterns of inhibition seen for potently neutralizing DIII-specific antibodies [18], [25], [46], [47].

### The E111 epitope is not accessible in the cryo-electron microscopy models of DENV

**Figure 5:**
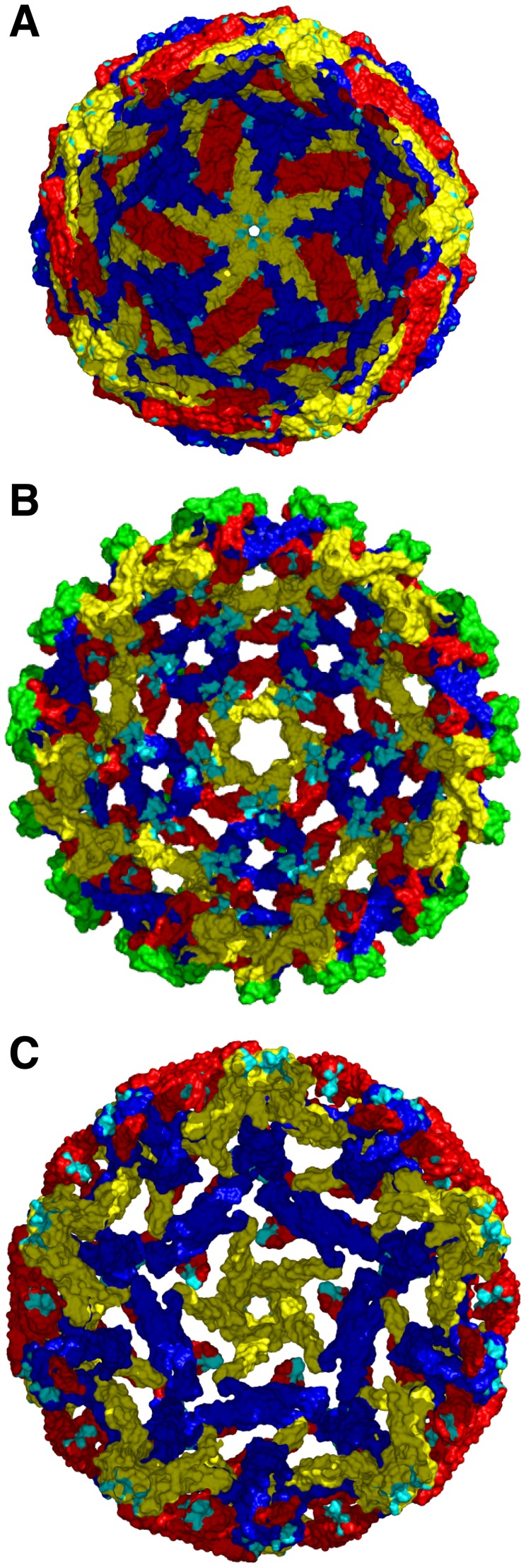
Mapping of the E111 epitope on the DENV virion. The atomic structures of the (**A**) mature (PDB 1K4R), (**B**) immature (PDB 3C6D), and (**C**) 1A1D-2-bound DENV-2 (PDB 2R6P) are shown as determined by modeling of cryo electron-microscopy reconstructions. E proteins in each icosahedral symmetry axis are highlighted: yellow (5-fold), blue (3-fold), or red (2-fold). The E111 epitope is colored in cyan in each symmetry group. To visualize the localization of the E111 epitope (on the interior of the viral surface in certain axes) a cross-section of each viral particle is shown. prM is colored in green on the immature virus model. In **panel C**, the 1A1D-2 Fabs were removed from the deposited cryo-electron microscopy structure to show only the antibody-stabilized virus conformation.

We next docked our E111-DIII structures onto the cryo-EM-derived model of the mature DENV virion [23]. With three envelope glycoproteins in the asymmetric unit, there are three potential E111-binding environments. However, in the mature DENV model, the E111 epitope was not accessible on the surface in any of the three symmetry environments (**Figure 5A**). Instead, the E111 epitope was buried in E protein contacts on the virion surface (**Figure S2A–E**).

Because some anti-flavivirus MAbs (e.g., DII fusion loop-specific) show differential neutralization of mature and partially mature virions, we hypothesized that intrinsic differences in particle maturation among different DENV-1 genotypes might impact neutralization by E111. However, the distinct neutralization profiles by E111 of DENV-1 16007 and West Pac-74 were not explained by differential epitope accessibility due to variation in the maturation state of the viruses (**Figure S3**). As in the mature virion model, the E111 epitope also appeared inaccessible on the cryo-EM model of the immature DENV virion [48], due to the trimeric arrangement of prM-E, which positions the epitope farther into the virus interior (**Figure 5B** and **Figure S2 F–H**).

The cryo-EM model of DENV-2 in complex with the 1A1D-2 Fab describes one conformational ensemble that is a consequence of “breathing” of a virus particle [31]. The 1A1D-2 epitope is partially inaccessible in the unbound conformation of the mature virion, and an increase in temperature allows for dissociation of E protein homodimers and greater exposure of the A-strand of DIII, a major component of the 1A1D-2 epitope. Although there are major rearrangements of the E proteins in this structure, E111 binding still would be prohibited by steric clashes of adjacent E protein monomers (**Figure 5C** and **Figure S2 I–K**) at the 3- and 5-fold axes of symmetry. While access of the 2-fold axis is not hindered by contacts with neighboring E proteins, its orientation would inhibit an immunoglobulin from binding this site. Based on these models, it appears unlikely that E111 binds to DENV-1 in the conformations that have been described by cryo-EM to date.

### Neutralization of DENV-1 by E111 varies with time and temperature in a genotype-dependent manner

**Figure 6:**
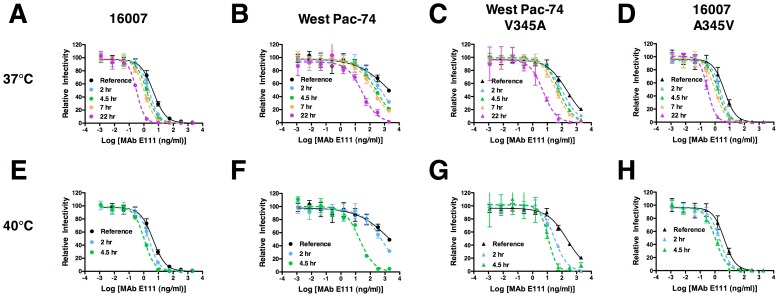
Neutralization of DENV-1 by E111 varies with time and temperature in a genotype-dependent manner. Serial dilutions of E111 were incubated with (**A** and **E**) DENV-1 16007, (**B** and **F**) West Pac-74, (**C** and **G**) V345A West Pac-74, or (**D** and **H**) A345V 16007 RVP for 1 hour at 37°C before the addition of Raji-DC-SIGNR cells to establish reference neutralization curves. Additional DENV-1 RVP-E111 complexes were incubated for 2, 4.5, 7, and 22 hours at 37°C (**A–D**) or 2 and 4.5 hours at 40°C (**E–H**) before addition to Raji-DC-SIGNR cells. Infection was carried out at 37°C and determined by flow cytometry 48 hours later. One representative experiment of three is shown. The data is normalized relative to the infectivity of the RVP in the absence of antibody at each time point for each temperature. Error bars indicate standard error of the mean of replicate infections.

Changes in time and temperature of binding can expose otherwise cryptic epitopes and enhance neutralizing activity of some MAbs [31], [32], [49]. Given our structural, biophysical, genetic, and virological data, we hypothesized that the CC′ epitope on West Pac-74 (genotype 4) was less well exposed compared to 16007 (genotype 2). Alternatively, a difference in the range of the ensemble structures sampled by the two viruses could contribute to the differential neutralization by E111. We compared the time- and temperature-dependence of neutralization of E111 with 16007 and observed little change in EC50 values after incubation of 16007 in the presence of antibody at 37°C or 40°C from 1 to 7 or 4.5 hours, respectively (**Figure 6A and E**); this suggests that the CC′ loop epitope generally is accessible among the ensemble of conformations sampled by 16007 under steady-state conditions. Similarly, a modest change in the pattern of neutralization was observed with E111 and West Pac-74 RVP after incubation at 37°C up through 7 hours (**Figure 6B**). However, we observed a marked increase in neutralization when E111 and West Pac-74 RVP were incubated at 40°C for 4.5 hours, with a 20-fold (P<0.001) reduction in the EC50 value (**Figure 6F**). By comparison, 16007 exhibited only a 3.5-fold fold increase in potency over the same interval. A shift in EC50 was observed with both 16007 and West Pac-74 after 22 hours at 37°C suggesting that over time, a greater number of E111 epitopes become exposed for binding (**Figure 6A and B**).

Because our SPR binding and structural data (**Figures 1** **and** **2**) did not correlate with neutralization experiments in which amino acids of 16007 and West Pac-74 were exchanged (**Figure 3**), we speculated that the interaction between E111 and amino acid 345 on DIII might be modulated by epitope accessibility in a genotype-dependent manner. We evaluated the effects on time and temperature on E111 neutralization of the reciprocal pair of DENV-1 RVP, V345A West Pac-74 and A345V 16007. We observed enhanced E111 neutralization of V345A West Pac-74 as a function of increased time and temperature (**Figure 6C and G**); by 22 hours at 37°C or 7 hours at 40°C, the EC50 value of V345A West Pac-74 RVP neutralization approached that of the wild type 16007 RVP (**Figure S4**). Neutralization of the A345V 16007 RVP by E111 also increased with time and temperature (**Figure 6D and H**), although there was no difference in EC50 value compared with wild type 16007 RVP. Overall, these experiments suggest that under steady-state conditions, the ensemble of structures with respect to exposure of the CC′ loop epitope are different between strains 16007 and West Pac-74. Virion conformations sampled by individual DENV-1 genotypes likely vary with temperature and differ from those described in existing cryo-electron microscopy models.

## Discussion

Antibody neutralization of flaviviruses requires multiple antibodies to bind a single virion until a neutralization threshold is reached. The ability of a MAb to bind a given viral epitope depends on its concentration, the affinity of its interaction with the infectious virus particle, and the accessibility of the epitope on the virion [11]. While some epitopes are readily accessible on the surface of mature DENV, others are partially or completely inaccessible [27], [29], [31], [50]. However, antibodies that recognize partially or completely occluded sites on the mature virion can still neutralize flavivirus infection because of particle heterogeneity with respect to maturation [29], [50] and/or by sampling of alternate ensemble structures or “breathing”, which allows for intermittent display of cryptic epitopes [31], [32]. Here, our structural studies show that E111 binds to a novel CC′ loop epitope on DIII that does not appear to be affected by particle maturation. Although the CC′ epitope is predicted to be inaccessible on both the mature and immature virion, E111 still potently neutralizes some but not all DENV-1 genotypes. While the amino acid sequence of DIII varies among genotypes in and around the CC′ loop, which affects E111 binding by SPR, there was a limited relationship between the kinetics of binding *in vitro* and the potency of genotypic neutralization in cell culture. Thus, some aspect of E111 recognition and neutralization appears independent of the epitope sequence. While E111-mediated neutralization of strain 16007 was less affected by changes in time or temperature of incubation, neutralization of West Pac-74 was enhanced substantially after incubation with E111 at higher temperatures and for longer times. These experiments suggest that at steady state, DENV-1 16007 has a broader ensemble of conformations compared to West Pac-74, allowing for enhanced exposure of particular DIII-specific epitopes for MAb neutralization. This phenomenon could explain in part why so many (13 of 15) of our DIII-specific MAbs strongly neutralized infection of strain 16007 but not West Pac-74 despite the relatively few amino acid changes in DIII [17]. Several of our other anti-DENV-1 MAbs map to the lateral ridge epitope on DIII, which should be fully exposed on the virion [51], and, in principle, not require temperature or time-dependent changes in structure for enhanced epitope accessibility. Nonetheless, in on-going studies, DIII lateral ridge epitope-specific MAbs (e.g., DENV1-E102, DENV1-E103, DENV1-E105, and DENV1-E106) all neutralized infection by DENV-1 West Pac-74 more efficiently after an increase of temperature and duration of incubation (K. Dowd and T. Pierson, unpublished results). Thus, for DENV-1, structural perturbations to the virion may influence neutralization by MAbs recognizing ostensibly more and less exposed epitopes in a strain-dependent manner.

Within DIII of different DENV-1 genotypes, the greatest sequence variation occurs within and surrounding the CC′ loop (4 of 9 sites). In comparison, the CC′ loop residues of other DENV serotypes are highly conserved: for DENV-2 and DENV-3 genotypes, only 2 of 8 and 1 of 11 sites, respectively, show amino acid variation within or proximal to the CC′ loop. Neutralizing antibodies that localize to the CC′ loop are not restricted to DENV-1. We recently mapped four inhibitory DENV-2 MAbs to residues within the CC′ loop by yeast surface display [18]. Several of our DENV-2-specific CC′ loop MAbs protected against DENV-2 challenge both as pre-exposure prophylaxis and post-exposure therapy in mice. Due to the lack of a reproducible mouse model for DENV-1 16007 infection, we have not assessed directly the therapeutic efficacy of E111 under conditions where it is highly neutralizing. Nonetheless, E111 was effective as prophylaxis against DENV-1 West Pac-74 in an immunocompromised AG129 mouse model of infection [17], despite its relatively poor EC50 value in cell culture.

Flavivirus virions can undergo structural re-arrangements with an increase of temperature, which can facilitate binding of antibodies to epitopes with limited accessibility [31], [32]. Indeed, DENV-1 RVP showed markedly enhanced neutralization by E111 that was dependent on both time and temperature of incubation with antibody. While these pre-incubation conditions alone improved neutralization of West Pac-74 RVP by E111, insertion of the V345A substitution (from 16007 into West Pac-74) was required to shift the neutralization curve to achieve an EC50 value of wild type 16007. This observation is consistent with a role for amino acid 345 in E111 engagement and correlates with differences in the binding of V345A and wild type DIII of West Pac-74 observed by SPR. Thus, while a lack of E111 epitope accessibility explains why West Pac-74 was not efficiently neutralized under steady-state conditions, prolonged time and higher temperature of incubation promoted sampling of a broader ensemble of structures that revealed the differential effect of residue 345 on neutralization of West Pac-74. Interestingly, the reciprocal mutation, A345V, when substituted into 16007 had essentially no impact on neutralization by E111, regardless of the time and temperature of incubation. While wild type DIII of 16007 binds E111 with a 37-fold longer half-life than the A345V variant, the on-rates were equivalent. Thus, E111 binding and neutralization may be preferentially determined by the on-rate kinetics of antibody attachment through stabilization of a potentially transient/infrequent conformation present in the 16007 ensemble of structures. Indeed, a mutant DIII of 16007 (K343I), which showed a substantially enhanced half-life of binding interaction (∼45 minutes) with E111, did not affect neutralization potency (S. K. Austin, M. Diamond, and D. Fremont, unpublished results).

Currently, there are no cryo-electron microscopy models of DENV-1, whereas several models of DENV-2 have been described [23], [31], [52]. These models were used as surrogates of DENV-1 in an attempt to understand how E111 engaged its epitope in the context of a virion. Due to the packing of individual E protein monomers in the particle, there are limitations of accessibility of antibodies to portions of the E protein depending upon its particular symmetry environment. Examination of the available cryo-electron microscopy models of DENV failed to explain how E111 binds to the CC′ loop on the virion, as it is completely inaccessible in all models, in all symmetry environments. DENV-2 particles are believed to sample ensemble of conformations [32], as shown in the captured intermediate of the cryo-electron microscopy reconstruction of DENV-2 with the 1A1D-2 Fab [31]. Despite a sizable increase in the relative E protein surface area exposed in the 1A1D-2 captured intermediate, from a structural perspective there was still insufficient accessibility to allow engagement by E111. Our crystallographic, kinetic, and functional data all support a role for the CC′ loop in E111 recognition yet the existing atomic models cannot explain how it engages the virion. We speculate that a particular structural ensemble allows exposure of the CC′ loop and binding of E111 for certain DENV-1 genotypes. Indeed, we know little about the alternate conformational states sampled by flaviviruses, as only two cryo-electron microscopy models of transitional flavivirus states exist: a low pH model of WNV E16 Fab and WNV, and the 1A1D-2 Fab binding to DENV-2 at physiological pH [31], [53]. Further investigation using antibody captured virus conformations are needed to explore the breadth of structures sampled by flaviviruses.

Our structural and functional characterization of E111 has implications for vaccine development and assessment. While natural infection with DENV is believed to confer durable protective immunity against homologous DENV serotypes, several papers have reported disparate neutralization titers of homotypic strains and genotypes after natural infection or immunization. The neutralization potency of patient sera during the course of an DENV-3 epidemic varied substantially for DENV-3 strains corresponding to distinct genotypes [33]. A study of sera from individuals experiencing DENV-1 infections also showed variable neutralizing activity against different DENV-1 strains [35]. Moreover, pooled sera from monkeys immunized with a tetravalent chimeric live attenuated DENV vaccine revealed a range (e.g., ∼12-fold for DENV-1 strains) of variability in EC50 neutralization titers against individual strains of a given DENV serotype [38]. It remains possible that the differences are even larger, as full neutralization profiles or EC90 values were not reported in this latter study.

Studies examining how genotypic variation affects neutralization with MAbs [16]–[18], [54], [55] suggest that natural sequence variation among genotypes of a DENV serotype impacts the potency of antibody neutralization. Analogously, many neutralizing antibodies against HIV, influenza, and hepatitis C viruses fail to inhibit related stains and/or serotypes [56]. While the cryptic nature of the CC′ loop may be a special case [17], we propose that disparate neutralization of DENV-1 strains by monoclonal or polyclonal antibodies could be due to or at least be affected by differences in the ensemble of conformations sampled by the virion. Selection of DENV strains that sample a greater diversity of conformations as vaccine candidates could broaden the repertoire of neutralizing antibodies against DENV. Such strains could better expose and present the spectrum of epitopes available, and thereby induce a more diverse neutralizing antibody repertoire. Alternatively, the use of DENV strains or formulations with a limited structural ensemble could focus the neutralizing antibody response on specific epitopes whose accessibility is independent of time and temperature, and thus, more effective at neutralizing a diverse range of strains, regardless of particle conformation. Although a monovalent formalin-fixed DENV-2 vaccine induced strongly neutralizing antibodies against the parent strain in mice and monkeys, it was never evaluated for activity against a range of strains corresponding to different genotypes [57]. Clearly, further empirical studies are necessary to assess directly how virion ensembles affect immunogenicity as well as pathogenesis. Finally, the conformational diversity of DENV strains used for diagnostic evaluation of polyclonal serum could affect the interpretation of its neutralizing potential; for example, the choice of a DENV strain that cycles through limited structural conformations at 37°C for neutralization assays could underestimate the quality of the inhibitory activity of the antibody response in human serum.

In summary, we have defined a novel structural epitope on the CC′ loop of DIII of DENV, which is not accessible in the existing cryo-electron microscopy reconstruction models of DENV particles. Our experiments also suggest that the ensemble of conformations of the DENV virion structure varies in a genotype-dependent manner, which impacts the neutralizing activity of antibodies and has direct implications for the development and analysis of candidate DENV tetravalent vaccines.

## Materials and Methods

### Protein production, purification, and crystallization

An untagged form of DENV-1 DIII (strain 16007, residues 293 to 399) was cloned into the pET21a vector (Novagen) and expressed by autoinduction [58] in BL21 bacterial cells (Agilent). Isolated inclusion bodies were solubilized and oxidatively re-folded, as previously described [59]. Variants of the DENV-1 16007 strain (residues 293–399) were generated by site-directed mutagenesis (QuikChange, Agilent) using unique primer sets (**Table S2**). E111 scFv was engineered with a (GGGGS)_3_ linker between the V_L_ and V_H_ and domains and a C-terminal hexahistadine tag, cloned into the pAK400 vector, and expressed in the periplasm of bacteria. The bacteria were lysed and the E111 scFv was purified by nickel affinity and size exclusion chromatography. The scFv was complexed with excess DIII and purified by size exclusion chromatography. The E111 scFv-DIII complexes were crystallized at 10 mg/ml by sitting-drop vapor diffusion at 20°C using 20% polyethylene glycol (PEG) 3350, 0.2 M potassium sulfate, and 5% glycerol. Crystals were cryo-protected in a solution containing 35% glycerol and cooled in liquid nitrogen.

After protein A affinity purification, the E111 IgG was cleaved with immobilized papain (Pierce Biotechnology), and Fabs were recovered, as the Fc and uncleaved IgG were removed by passage over a second protein A affinity column. West Pac-74 DIII and E111 Fab were mixed and isolated by size exclusion chromatography on a S75 Superdex column. The E111 Fab-DIII complexes were crystallized at 15.8 mg/ml by sitting-drop vapor diffusion at 20°C using 0.1 M MES pH 5.3, 20% PEG 6000 (final pH 6.0) with 1% glycerol. The crystals were cryo-protected in the mother solution supplemented with 20% ethylene glycol and cooled in liquid nitrogen.

### Structure determination and refinement

Data were collected at APS beamline 19–ID (Argonne National Laboratories) at 293° K and at a wavelength of 1.007 Å using a CCD detector. Data were processed, scaled, and merged with HKL-2000 [60]. Crystallographic phasing for the E111 scFv-DIII complex was obtained by molecular replacement (PHENIX [61]) using the predicted scFv model given by the PIGS server [62] and the atomic structure of DENV-1 16007 DIII (PBD accession number 3IRC [17]). The crystals belong to the space group P4_3_2_1_2 with the unit cell dimensions of a = b = 135.224 and c = 52.221, with one E111 scFv-DIII complex per asymmetric unit. An atomic model was iteratively built in COOT [63] and refined in PHENIX, and contained 328 amino acids (residues 298–396 from DIII, 1–114 of the E111 V_H_, and 1–107 of the E111 V_L_, (Chothia numbering), 147 water molecules, four chloride ions and one sulfate and glycol molecule each. The final 2.5 Å resolution model was refined to an R_work_ = 19.6% and R_free_ = 23.9% for all F>0, with excellent geometry and Ramachandran angles (97.4% favored and 0.3% outliers).

Data for the E111 Fab-West Pac-74 DIII complex were initially processed with centered orthorhombic symmetry with subsequent identification of pseudo-merohedral twinning. The crystals actually belong to space group P2_1_ and suffer ∼30% twinning with the operator h,-k,-h-l. The data was successfully phased by molecular replacement using the E111 scFv-DIII complex and the constant domains from PDB ID 4AEH with two molecules per asymmetric unit. The atomic model was iteratively build in COOT and refined in REFMAC [64] and PHENIX using jelly body and reference model restrains, respectively. The structure contained 1060 amino acids (residues 299–395 from DIII, 1–212 from the light chain, and 1–212 from the heavy chain (Chothia numbering). The final 3.8 Å resolution model was refined to an R_work_ = 23.7% and R_free_ = 27.8% for all F>0, with excellent geometry and Ramachandran angles (97.0% favored and 0.4% outliers). The atomic coordinates and structure factors have been deposited in the Protein Data Bank (www.rcsb.org) under accession numbers 4FFY and 4FFZ for the scFv and Fab complexes, respectively.

### Surface plasmon resonance

Kinetic information on the interaction between E111 and DIII variants was obtained using a Biacore T100 instrument. Approximately 500 response units (RU) of E111 or control MAb/scFv (WNV E16) was immobilized using amine coupling to a Series S CM5 chip. Once stabilized, a two-fold dilution series of the DENV DIII variants were injected over the chip at a flow rate of 65 ml/min for 180 seconds and allowed to dissociate for 1,000 seconds. DIII had dissociated over this time period and additional regeneration was not necessary. Data was processed using the Biacore Evaluation Software (Version 1.1.1) by double referencing and a 1∶1 Langmuir fit of the curves. All curves were reference subtracted from a flow cell containing the negative control WNV E16 MAb/scFv. Maximum response units were plotted versus concentration and this curve was fitted to determine the *K*_D_. Results were generated from at least three independent experiments, with a minimum of six binding curves per experiment.

### Plaque reduction neutralization tests (PRNT)

PRNT were performed with the five DENV-1 genotype strains with E111 on Vero cells as described previously [17]. In some experiments, pre- or post-attachment studies were performed as a variation [18], [46]. Briefly, serially diluted MAbs were mixed 1∶1 with 10^2^ PFU of 16007 DENV-1 virus in DMEM containing 10% FBS and incubated for one hour at 4°C. The virus-MAb mixture was then added to the cells at 4°C, and after washing, incubated at 37°C for one additional hour. Alternatively, cells and media were chilled to 4°C before 10^2^ PFU of virus was added and incubated for one hour. Unbound virus was washed away with chilled media before the addition of E111 MAb. After one hour at 4°C, cells were washed with warm media and overlaid with 2% low-melt agarose (SeaPlaque) in modified Eagle medium and 4% FBS and incubated at 37°C for 6 days. PRNT50 values were determined using non-linear regression analysis (Graph Pad Prism4).

### MAb neutralization of DENV-1 using RVP

DENV-1 RVP were generated as described previously [32], [41]. Plasmids expressing the wild type or mutant capsid (C)-prM-E genes of DENV-1 (strain 16007 or West Pac-74) were co-transfected into HEK293T cells with a plasmid encoding a sub-genomic WNV replicon expressing GFP. E protein variants were engineered by site-directed mutagenesis (QuikChange, Agilent) and confirmed by sequencing. Standard neutralization assays with RVP were performed by incubating serial dilutions of antibody with DENV-1 RVP for 1 hour at 37°C, followed by addition of Raji-DC-SIGNR cells. Infection was carried out at 37°C and monitored by flow cytometry 48 hours later for GFP expression. To assess the role of temperature on MAb activity, neutralization assays were performed as above, and designated as “reference” neutralization profiles. Additional RVP-antibody complexes, following the initial 1 hour incubation at 37°C, were further incubated at 37°C or 40°C for incremental lengths of time, followed by infection of Raji-DC-SIGNR cells. Relative infectivity was determined after comparison to infectivity of DENV-1 RVP incubated at the same temperature in parallel in the absence of antibody.

### Effects of maturation on E111 MAb neutralization

RVP were produced from HEK293T cells to represent various stages of maturation (standard (containing a heterogeneous mixture of partially mature and mature) or mature (produced in the presence of an over-expression of furin)) according to published protocols [29]. Standard neutralization assays with RVP were performed by incubating serial dilutions of antibody with DENV-1 RVP for 1 hour at 37°C, followed by addition of Raji-DC-SIGNR cells. Infection was carried out at 37°C and monitored by flow cytometry 48 hours later for GFP expression. To assess the role of temperature on MAb activity, neutralization assays were performed as above, and designated as “reference” neutralization profiles.

### Docking of E111 scFv onto structural models

*(a) E protein dimer*. Docking of the E111-DIII structure and the WNV E16 Fab-DIII (PDB 1ZTX) onto the pre-fusion dimer structure of DENV2 (PDB 1OAN) was based upon superimposition of DIII. *(b) E protein trimer*. The same procedure was used for docking of the E111 scFv onto the post-fusion DENV-1 trimer structure (PDB 3G7T). *(c) Virions*. The coordinates for the full mature (PDB 1KR4), immature (PDB 3C6D), and 1A1D-2-bound (PDB 2R6P) DENV-2 virus assemblies were downloaded from VIPERdb [65] (http://viperdb.scripps.edu/). The surface of the virus was clipped to reveal the interior of the virion models. All structural representations were colored and rendered using PyMOL (The PyMOL Molecular Graphics System, Version 1.4–1.5.1 Schrödinger, LLC., http://www.pymol.org).

## Supporting Information

Figure S1 **Expression and purification of proteins.** (**A**) A scheme of E111 scFv construct design, expression, and purification with DIII of 16007. (**B**) A scheme of the proteolytic cleavage of the E111 IgG2c molecule, purification, and complex purification with DIII of West Pac-74. (**C**) Structural alignment of ribbon representations of the two structures. The chain colors are as follows: 16007 DIII (white), scFv light variable domain (cyan), scFv heavy variable domain (magenta), and the E111 Fab-West Pac-74 structure is in light green. (**D**) Detailed hydrogen bonding interactions of 16007 DIII CC′ loop residues (yellow) with E111 light (cyan) and heavy (magenta) chains, with interfacial waters (red) evident on the composite electron density omit map.
(PDF)

Figure S2 **The E111 epitope is occluded in the three existing cryo-electron microscopic models of DENV for different reasons.** The equivalent residues were mapped onto the surface of the DENV-2 mature cryo-electron microscopy atomic reconstruction model (see **Figure 5A**; PDB 1K4R). The orientation of bound E111 places the Fab within the plane of the E protein arrangement on the viral surface (**Figure S2A**), DIII in gold at the 3-fold axis. The contacts made by the E111 scFv (**Figure S2B**, colored as in **Figure 1D**) are shown in contrast to the contacts made by neighboring E proteins for the mature virus in the two-fold (**C**), three-fold (**D**), or five-fold (**E**) axes of symmetry. The DIII molecules are oriented and colored as in **Figure 1D**, while the contacts of the adjacent E proteins are shown in red. The immature form of DENV-2 (PDB 3C6D) shows a different impediment to E111 engagement. The formation of the prM-E heterotrimers on the surface of the virus pushes the E111 epitope towards the interior of the virus (see **Figure 5B**). Individual chains from the 2-fold (red), 3-fold (blue), and 5-fold (yellow) associated with prM form the homotrimeric spikes on the surface of the immature virus (**Figure S2F–H**). While the E proteins from each chain contact at DIII (**H**), the repositioning of DIII towards the interior of the immature virus (**F**, *side view*, and **G**, *bottom view*) prevents its accessibility in this model. The model of DENV complexed with the 1A1D-2 Fab (PDB 2R6P) is shown in **Figure 5C**. The E111 epitope is surface accessible in all three axes of symmetry, in contrast to that of the immature virus. However, steric hindrance at the 3-fold (**I**) and 5-fold (**J**) axes due to the tight spatial arrangement of neighboring DIII prohibits E111 engagement. Adjacent E proteins do not contact DIII at the 2-fold axis. However, due to the orientation of the E111 epitope at this axis, there is insufficient space for an intact IgG molecule to bind (**K**, looking towards the center of the virus).
(PDF)

Figure S3 **E111 neutralization occurs independently of the maturation state of DENV-1 particles.** **A–D**. Serial dilutions of (**A–B**) E60 (DII-fusion loop) or (**C–D**) E111 (DIII CC′ loop) were added to the heterogeneous mixture of DENV-1 RVPs released from cells using standard production conditions (*std, green triangles*) or a more homogeneous mature population (*mat, blue circles*) of (**A and C**) DENV-1 West Pac-74 or (**B and D**) DENV-1 16007 RVPs to determine the effect of the virus maturation state on MAb neutralization. MAb-RVP complexes were incubated for one hour at 37°C before being added to Raji-DC-SIGNR cells. Infectivity was determined by flow cytometry 48 hours later. One representative experiment of three is shown. The data is normalized relative to the infectivity of the RVPs in the absence of antibody. Error bars indicate standard error of the mean of replicate infections.
(PDF)

Figure S4 **Neutralization potency of DENV-1 by E111 for V345A West Pac-74 RVPs approaches that of wild type 16007 RVPs with an increase of time and temperature.** Serial dilutions of E111 were incubated at (**A**) 37°C or (**B**) 40°C with DENV-1 16007, West Pac-74, and V345A West Pac-74 RVPs for 1 hour or 22 hours before the addition of Raji-DC-SIGNR cells to establish reference neutralization curves. Infection was carried out at 37°C and determined by flow cytometry 48 hours later. One representative experiment of three is shown. The data is normalized relative to the infectivity of the RVPs in the absence of antibody at each time point for each temperature. Error bars indicate standard error of the mean of replicate infections.
(PDF)

Table S1 **E111-DIII interface.**
(PDF)

Table S2 **DENV-1 DIII oligonucleotide primers.**
(PDF)

Text S1 **Supplemental methods and materials and supplemental references.**
(DOCX)
