# Supplementary figures and images for: Structural Basis of Differential Neutralization of DENV-1 Genotypes by an Antibody that Recognizes a Cryptic Epitope
Source: PLoS Pathog. 2012 Oct 4;8(10):e1002930. doi: 10.1371/journal.ppat.1002930 (PMC3464233; doi:10.1371/journal.ppat.1002930)

**A**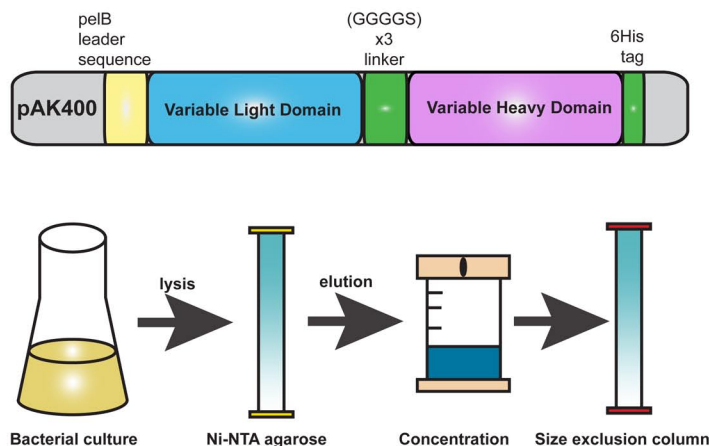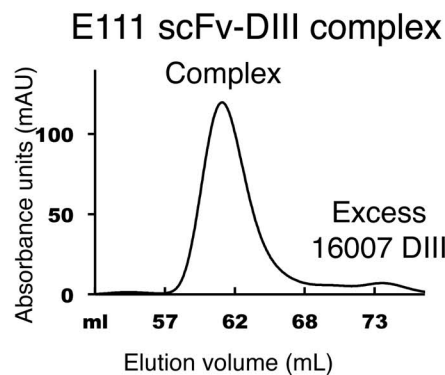**B**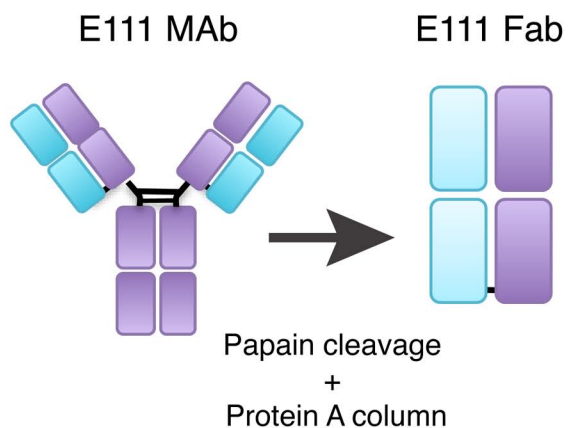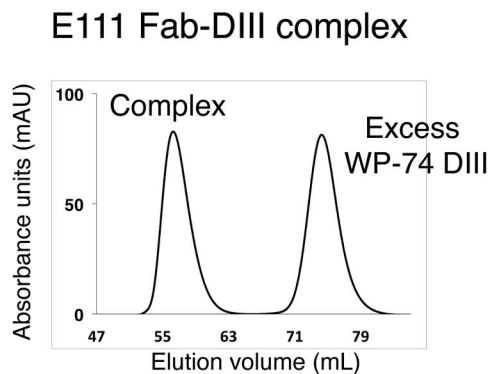**C**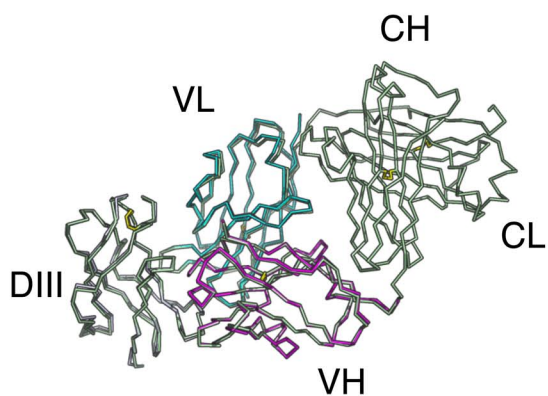**D**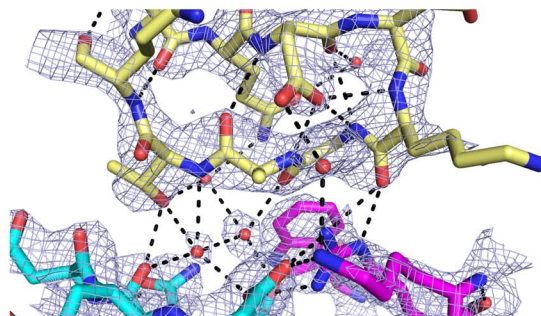**Figure S1**

Supplement: Figure S1 — Expression and purification of proteins. (A) A scheme of E111 scFv construct design, expression, and purification with DIII of 16007. (B) A scheme of the proteolytic cleavage of the E111 IgG2c molecule, purification, and complex purification with DIII of West Pac-74. (C) Structural alignment of ribbon representations of the two structures. The chain colors are as follows: 16007 DIII (white), scFv light variable domain (cyan), scFv heavy variable domain (magenta), and the E111 Fab-West Pac-74 structure is in light green. (D) Detailed hydrogen bonding interactions of 16007 DIII CC′ loop residues (yellow) with E111 light (cyan) and heavy (magenta) chains, with interfacial waters (red) evident on the composite electron density omit map. (PDF) [file ppat.1002930.s001.pdf]

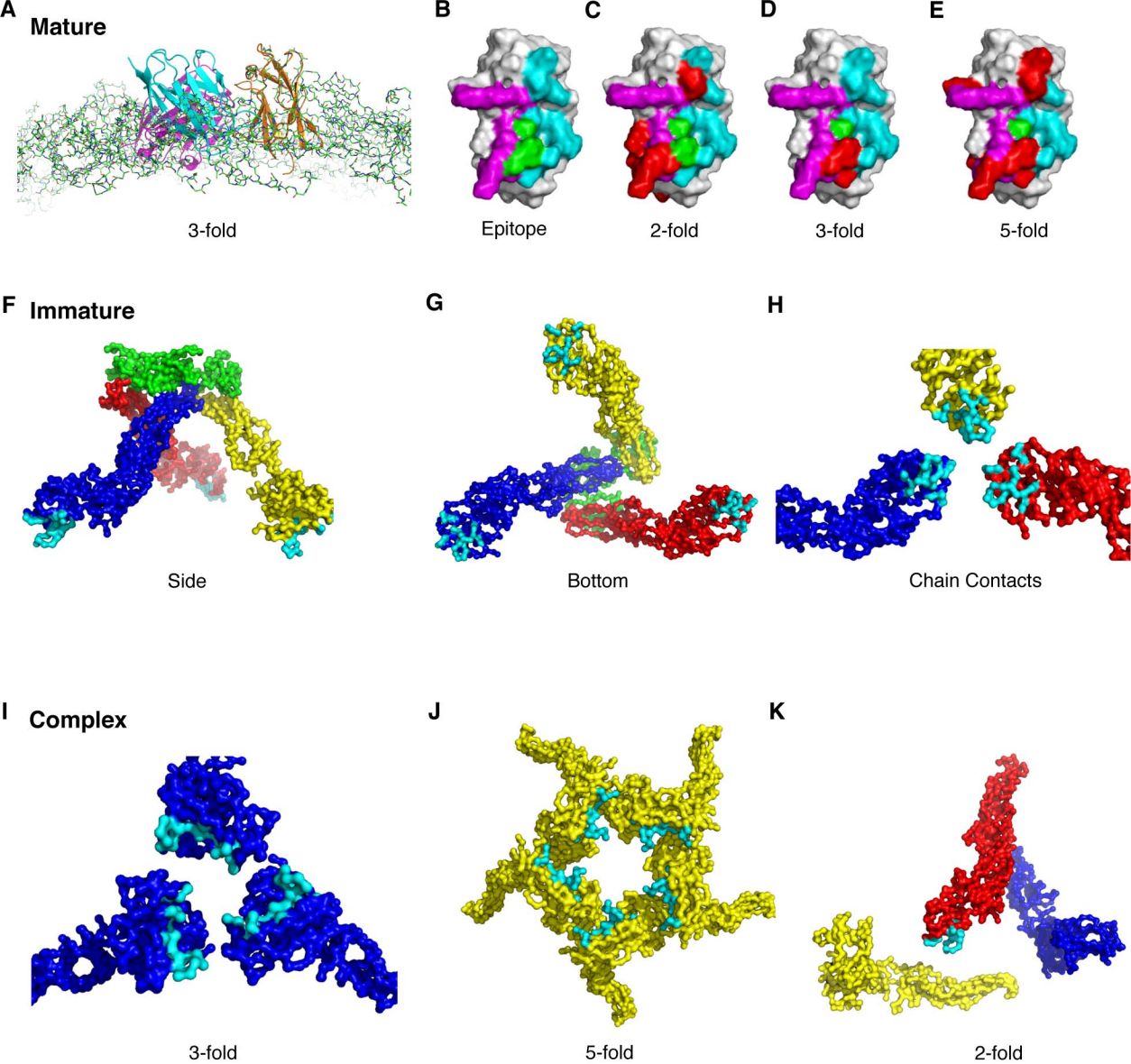

**Figure S2**

Supplement: Figure S2 — The E111 epitope is occluded in the three existing cryo-electron microscopic models of DENV for different reasons. The equivalent residues were mapped onto the surface of the DENV-2 mature cryo-electron microscopy atomic reconstruction model (see Figure 5A ; PDB 1K4R). The orientation of bound E111 places the Fab within the plane of the E protein arrangement on the viral surface (Figure S2A), DIII in gold at the 3-fold axis. The contacts made by the E111 scFv (Figure S2B, colored as in Figure 1D ) are shown in contrast to the contacts made by neighboring E proteins for the mature virus in the two-fold (C), three-fold (D), or five-fold (E) axes of symmetry. The DIII molecules are oriented and colored as in Figure 1D , while the contacts of the adjacent E proteins are shown in red. The immature form of DENV-2 (PDB 3C6D) shows a different impediment to E111 engagement. The formation of the prM-E heterotrimers on the surface of the virus pushes the E111 epitope towards the interior of the virus (see Figure 5B ). Individual chains from the 2-fold (red), 3-fold (blue), and 5-fold (yellow) associated with prM form the homotrimeric spikes on the surface of the immature virus (Figure S2F–H). While the E proteins from each chain contact at DIII (H), the repositioning of DIII towards the interior of the immature virus (F, side view, and G, bottom view) prevents its accessibility in this model. The model of DENV complexed with the 1A1D-2 Fab (PDB 2R6P) is shown in Figure 5C . The E111 epitope is surface accessible in all three axes of symmetry, in contrast to that of the immature virus. However, steric hindrance at the 3-fold (I) and 5-fold (J) axes due to the tight spatial arrangement of neighboring DIII prohibits E111 engagement. Adjacent E proteins do not contact DIII at the 2-fold axis. However, due to the orientation of the E111 epitope at this axis, there is insufficient space for an intact IgG molecule to bind (K, looking towards the center of the virus). (PDF) [file ppat.1002930.s002.pdf]

**A**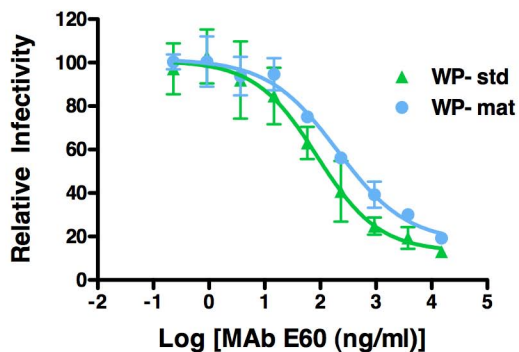**B**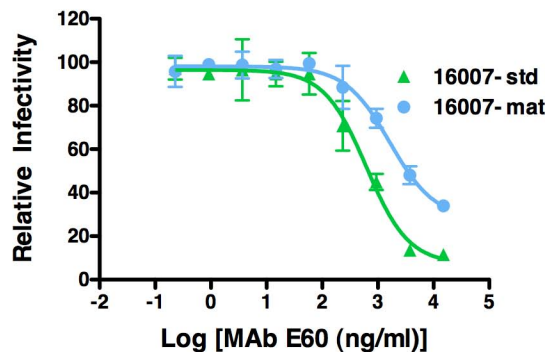**C**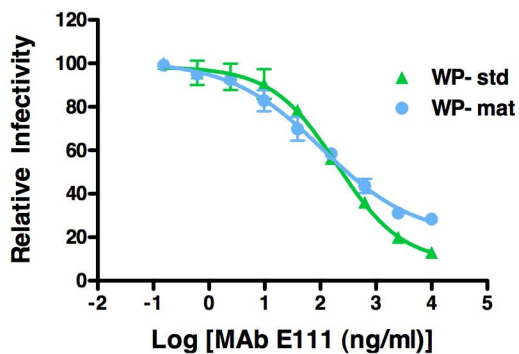**D**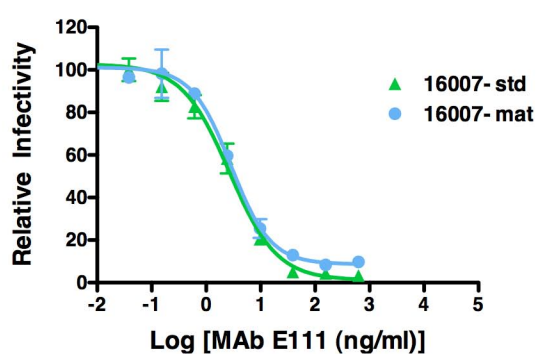**Figure S3**

Supplement: Figure S3 — E111 neutralization occurs independently of the maturation state of DENV-1 particles. A–D. Serial dilutions of (A–B) E60 (DII-fusion loop) or (C–D) E111 (DIII CC′ loop) were added to the heterogeneous mixture of DENV-1 RVPs released from cells using standard production conditions (std, green triangles) or a more homogeneous mature population (mat, blue circles) of (A and C) DENV-1 West Pac-74 or (B and D) DENV-1 16007 RVPs to determine the effect of the virus maturation state on MAb neutralization. MAb-RVP complexes were incubated for one hour at 37°C before being added to Raji-DC-SIGNR cells. Infectivity was determined by flow cytometry 48 hours later. One representative experiment of three is shown. The data is normalized relative to the infectivity of the RVPs in the absence of antibody. Error bars indicate standard error of the mean of replicate infections. (PDF) [file ppat.1002930.s003.pdf]

**A****37°C****Relative Infectivity**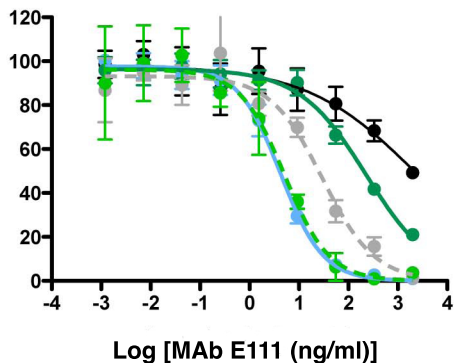**B****40°C****Relative Infectivity**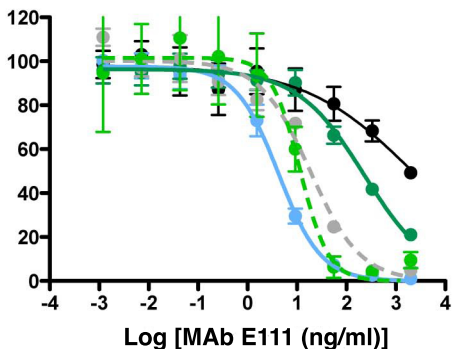**Figure S4**

Supplement: Figure S4 — Neutralization potency of DENV-1 by E111 for V345A West Pac-74 RVPs approaches that of wild type 16007 RVPs with an increase of time and temperature. Serial dilutions of E111 were incubated at (A) 37°C or (B) 40°C with DENV-1 16007, West Pac-74, and V345A West Pac-74 RVPs for 1 hour or 22 hours before the addition of Raji-DC-SIGNR cells to establish reference neutralization curves. Infection was carried out at 37°C and determined by flow cytometry 48 hours later. One representative experiment of three is shown. The data is normalized relative to the infectivity of the RVPs in the absence of antibody at each time point for each temperature. Error bars indicate standard error of the mean of replicate infections. (PDF) [file ppat.1002930.s004.pdf]
