# Supplementary material for: Structural Basis of Differential Neutralization of DENV-1 Genotypes by an Antibody that Recognizes a Cryptic Epitope
Source: PLoS Pathog. 2012 Oct 4;8(10):e1002930. doi: 10.1371/journal.ppat.1002930 (PMC3464233; doi:10.1371/journal.ppat.1002930)
Supplement: Table S1 — E111-DIII interface. (PDF) [file ppat.1002930.s005.pdf]

**Table S1. E111-DIII interface**

| 16007 DIII – E111 scFv | | | Western Pacific-74 DIII – E111 Fab | |
|---|---|---|---|---|
| **Van der Waals contacts[a]** | | | | |
| **DIII** | **E111 scFv** | | **E111 Fab** | |
| Val300 | AsnL53 | | | |
| Met301 | AsnL53 | | AsnL53<br>LeuL50 | |
| Lys334 | AsnL30D | | AsnL30D | |
| Pro336 | TrpH99 | | TrpH99 | |
| Phe337 | TyrL30B | | TyrL30B | |
| Ser338 | HisL30A<br>ProH98 | | PheL32<br>HisL30A<br>ProH98 | |
| Thr339 or<br>Ser339 | HisL30A<br>TyrL30B | | HisL30A<br>TyrL30B | |
| Gln340 | TrpH95<br>PheH97<br>ProH98 | | PheH97<br>ProH98 | |
| Glu342 | ValH56 | | | |
| Lys343 | TrpH33<br>ArgH50<br>ValH56<br>AspH57<br>LysH58 | | TrpH33<br>ArgH50<br>AspH57 | |
| Gly344 | ArgH50<br>TrpH95 | | ArgH50<br>TrpH95 | |
| Ala345<br>or<br>Val345 | AspL94<br>ArgH50 | | AspL94<br>ArgH50<br>LysH58 | |
| Thr346 | HisL30A<br>AsnL91<br>AsnL92 | | HisL30A<br>AsnL91<br>AsnL92 | |
| Gln347 | HisL30A | | HisL30A | |
| Gly349 | TyrL30B | | TyrL30B | |
| Arg350 | TryL30B | | TyrL30B | |
| Leu351 | TyrL30B | | TyrL30B | |
| Pro372 | TyrL30B | | TyrL30B | |
| Val379 | PheH97 | | PheH97 | |
| Ala382 | PheH96<br>PheH97<br>TrpH99 | | PheH96<br>PheH97<br>TrpH99 | |
| Gly383 | PheH96<br>PheH97 | | PheH96<br>PheH97 | |

| | | | | |
|---|---|---|---|---|
| Glu384 | AsnH31<br>TrpH32 | | AsnH31<br>TrpH32 | |
| | | | | |
| **E111/DIII putative hydrogen bonds[b]** | | | | |
| **DIII** | **E111<br>scFv** | **Distance (Å)** | **E111<br>Fab** | **Distance (Å)** |
| Thr/Ser339 (OG1) | TyrL30B (OH) | 2.96 | TyrL30B (OH) | 2.71 |
| Ser339 (O) | | | HisL30A (NE2) | 2.95 |
| Lys343(O) | ArgH50 (NE) | 2.93 | ArgH50 (NE) | 3.29 |
| Lys343(O) | ArgH50 (NH1) | 2.90 | ArgH50 (NH1) | 3.20 |
| Lys343(NZ) | AsnH57 (O) | 3.00 | AsnH57 (O) | 3.32 |
| Thr346 (OG1) | AsnL91 (O) | 2.77 | AsnL91 (O) | 2.81 |
| Gly383 (N) | PheH97 (O) | 2.59 | PheH97 (O) | 2.84 |
| | | | | |
| **E111/DIII water mediated hydrogen bonds[b,c]** | | | | |
| **DIII** | **E111** | | | |
| Ile335 (O) | AsnL53 (OD1) | | | |
| Phe337 (O) | AsnL30D (OD1)<br>HisL30A (O) | | | |
| Asp341 (OD2) | LysH58 (NZ) | | | |
| Lys343 (NZ) | AsnH57 (O)<br>AsnH57 (OD1) | | | |
| Gly344 (O) | TyrL96 (OH) | | | |
| Thr346 (O) | AspL92 (O) | | | |
| Thr346 (OG1/N) | AspL91 (O)<br>AsnL92 (O)<br>AsnL94 (OD1) | | | |
| Asn348 (OD1) | HisL30A (ND1)<br>AsnL92 (OD1) | | | |
| Asn348 (OD1) | AsnL92 (O) | | | |
| Gly349 (O) | TyrL30B (N) | | | |

[a]Van der Waals contacts have interatomic distance ≤ 4.0 Å

[b]Putative H-bonds defined by HBPlus (McDonald IK)

[c]Water molecules were not placed in the E111 Fab-West Pac-74 DIII structure.
