# Supplementary material for: Structural Basis of Differential Neutralization of DENV-1 Genotypes by an Antibody that Recognizes a Cryptic Epitope
Source: PLoS Pathog. 2012 Oct 4;8(10):e1002930. doi: 10.1371/journal.ppat.1002930 (PMC3464233; doi:10.1371/journal.ppat.1002930)
Supplement: Table S2 — DENV-1 DIII oligonucleotide primers. (PDF) [file ppat.1002930.s006.pdf]

Table S2. DENV-1 DIII oligonucleotide primers.

	DIII Construct Name	Primer sequence
1	Western Pacific-74	5’-GCAAGTTCCCTTTTCGAGCCAAGATGAGAAAGG-3’<br>5’-CCAAGATGAGAAAGGAGTAACCCAGAATGGGAGAT-3’
2	3146 SL	5’-GCATGACTTTAAAAGGGGTGTCATATGTGATGTGCACAG-3’<br>5’-GCAAGATTCCCTTTTCGACCCAAAATGAGAAAGGAGCAA-3’<br>5’-CCAAGATGAGAAAGGAGTAACCCAGAATGGGAGAT-3’<br>5’-GCCAACCCCATAGTCACTGACAGAGAAAAACCAGTC-3’<br>5’-CCAGTCAATATTGAGACAGAACCACCCTTTGGTGAG-3’<br>5’-GGTGAGAGCTACATCGTGATAGGAGCAGGTG-3’
3	TVP-2130	5’-CCAAGATGAGAAAGGAGTAACCCAGAATGGGAGAT-3’<br>5’-GGAGCAACCCAGAATGGGAGAGTAATAACAGCCAACCC-3’<br>5’-GTGCTCGAGCTACCCTATGGTGCTTCCTTTCTTGAACCA-3’
4	TVP-5175 (16007 A345I)	5’-CCCTTTTCGACCCAAGATGAGAAAGGAATAACCCAGAAT-3’
5	16007 K310E/T329E /K361T	5’-GCTCATTCAAGTTAGAGGAAGAAGTGGCTGAGACC-3’<br>5’-CTGGTGCAGGTTAAATATGAAGGAGAAGACGCACCATGC-3’<br>5’-CCAACCCCATAGTCACTGACACCGAAAAACCAGTCAATATT GAG-3’
6	16007 T339S	5’-GCAAGTTCCCTTTTCGAGCCAAGATGAGAAAGG-3’
7	16007 D341N	5’-GCAAGATTCCCTTTTCGACCCAAAATGAGAAAGGAGCA-3’
8	16007 A345V	5’-CCAAGATGAGAAAGGAGTAACCCAGAATGGGAGAT -3’
9	16007 L351V	5’-GGAGCAACCCAGAATGGGAGAGTAATAACAGCCAACC-3’
12	16007 A369T	5’-CCAGTCAATATTGAGACAGAACCACCCTTTGGTGAG-
13	16007 V380I	5’-GGTGAGAGCTACATCGTGATAGGAGCAGGTG -3’

Table of primers used for site-directed mutagenesis of DENV-1 16007 DIII.
