# Supplementary material for: Structural Basis of Differential Neutralization of DENV-1 Genotypes by an Antibody that Recognizes a Cryptic Epitope
Source: PLoS Pathog. 2012 Oct 4;8(10):e1002930. doi: 10.1371/journal.ppat.1002930 (PMC3464233; doi:10.1371/journal.ppat.1002930)
Supplement: Text S1 — Supplemental methods and materials and supplemental references. (DOCX) [file ppat.1002930.s007.docx]

**Supplemental Methods and Materials**

**Effects of maturation on E111 MAb neutralization.** RVP were produced from HEK293T cells to represent various stages of maturation (standard (containing a heterogeneous mixture of partially mature and mature) or mature (produced in the presence of an over-expression of furin)) according to published protocols [1]. Standard neutralization assays with RVP were performed by incubating serial dilutions of antibody with DENV-1 RVP for 1 hour at 37°C, followed by addition of Raji-DCSIGNR cells. Infection was carried out at 37°C and monitored by flow cytometry 48 hours later for GFP expression. To assess the role of temperature on MAb activity, neutralization assays were performed as above, and designated as “reference” neutralization profiles.

**Supplemental References**

1. Nelson S, Jost CA, Xu Q, Ess J, Martin JE, et al. (2008) Maturation of West Nile Virus Modulates Sensitivity to Antibody-Mediated Neutralization. PLoS Pathog 4: e1000060. doi:10.1371/journal.ppat.1000060.
